# Sigma-1 receptor expression in sensory neurons and the effect of painful peripheral nerve injury

**DOI:** 10.1186/1744-8069-9-47

**Published:** 2013-09-10

**Authors:** Madhavi L Bangaru, Dorothee Weihrauch, Qing-Bo Tang, Vasiliki Zoga, Quinn Hogan, Hsiang-en Wu

**Affiliations:** 1 Department of Anesthesiology, Medical College of Wisconsin, 8701 Watertown Plank Road, Milwaukee, WI 53226, USA; 2 Zablocki Veterans Administration Medical Center, Milwaukee, WI, USA

**Keywords:** Sigma-1 receptor, Neuropathic pain, Peripheral nerve injury, Endoplasmic reticulum, Sensory neuron, Dorsal root ganglion

## Abstract

**Background:**

The sigma-1 receptor (σ1R), an endoplasmic reticulum chaperone protein, is widely distributed and regulates numerous intracellular processes in neurons. Nerve injury alters the structure and function of axotomized dorsal root ganglion (DRG) neurons, contributing to the development of pain. The σ1R is enriched in the spinal cord and modulates pain after peripheral nerve injury. However, σ1R expression in the DRG has not been studied. We therefore characterized σ1R expression in DRGs at baseline and following spinal nerve ligation (SNL) in rats.

**Results:**

Immunohistochemical (IHC) studies in DRG sections show σ1R in both neuronal somata and satellite glial cells. The punctate distribution of σ1R in the neuronal cytoplasm suggests expression in the endoplasmic reticulum. When classified by neuronal size, large neurons (>1300 μm) showed higher levels of σ1R staining than other groups (700-1300 μm, <700 μm). Comparing σ1R expression in neuronal groups characterized by expression of calcitonin gene-related peptide (CGRP), isolectin-B4 (IB4) and neurofilament-200 (NF-200), we found σ1R expression in all three neuronal subpopulations, with highest levels of σ1R expression in the NF-200 group. After SNL, lysates from L5 DRGs that contains axotomized neurons showed decreased σ1R protein but unaffected transcript level, compared with Control DRGs. IHC images also showed decreased σ1R protein expression, in SNL L5 DRGs, and to a lesser extent in the neighboring SNL L4 DRGs. Neurons labeled by CGRP and NF-200 showed decreased σ1R expression in L5 and, to a lesser extent, L4 DRGs. In IB4-labeled neurons, σ1R expression decreased only in axotomized L5 DRGs. Satellite cells also showed decreased σ1R expression in L5 DRGs after SNL.

**Conclusions:**

Our data show that σ1R is present in both sensory neurons and satellite cells in rat DRGs. Expression of σ1R is down-regulated in axotomized neurons as well as in their accompanying satellite glial cells, while neighboring uninjured neurons show a lesser down-regulation. Therefore, elevated σ1R expression in neuropathic pain is not an explanation for pain relief after σ1R blockade. This implies that increased levels of endogenous σ1R agonists may play a role, and diminished neuroprotection from loss of glial σ1R may be a contributing factor.

## Background

Sigma-1 receptor (σ1R), a non-opioid receptor residing at the mitochondria-associated endoplasmic reticulum (ER) membrane [1], is widely distributed in various tissues including heart, liver, immune cells, as well as the nervous system [2-6]. Cellular functions are regulated by σ1R binding through a wide range of signaling pathways including protein kinases, ion channels, and transcription factors [7-10]. The σ1Rs are abundant in the central nervous system, where they may contribute to psychostimulant addiction [11,12], neurodegenerative disorders [13], neuroinflammation [14] and neuroprotection [15,16]. We have previously demonstrated that the σ1R mediates (+)-morphine induced anti-analgesia through actions at both supraspinal and spinal sites [17-19]. Other reports show that opioid ligand-induced antinociception is attenuated by σ1R agonists and potentiated by σ1R antagonists [20-23]. Further, σ1R antagonists effectively attenuate painful behavior in neuropathic and inflammatory pain models [24-29]. Although much remains to be learned about the role of the σ1R in health and disease, these various findings together point toward a significant role in pain modulation.

Peripheral nerve injury initiates multiple structural and functional changes in peripheral, spinal cord, and supraspinal sites that contribute to long-term neuropathic pain [30-34]. Our previous findings demonstrate that injured sensory neurons have diminished voltage-gated Ca^2+^ influx and reduced intracellular Ca^2+^ stores [35-37]. These features are of particular relevance to a potential role of σ1R in neuropathic pain since store depletion activates σ1R [1,38], and σ1R activation reduces *I*_Ca_ in other types of neurons [6,39]. Furthermore, the dorsal root ganglion (DRG), site of the somata of peripheral sensory neurons, generates neurosteroids that are σ1R ligands [40-42]. Although this evidence suggests that peripheral sensory neuron σ1Rs may contribute to the complex of changes that result in chronic neuropathic pain, expression of σ1Rs in this neuronal population has not been explored. In the present study, we therefore characterize the expression and distribution of σ1R in sensory neurons. Since elevated σ1R expression could contribute to the generation of neuropathic pain, we also examine the influence of painful nerve injury. We found that σ1Rs are present in both neurons and satellite glial cells (SGCs) in the DRG, and that σ1Rs are down-regulated after peripheral nerve injury.

## Results

A total of 31 rats were used in the present study, of which 16 were Control animals and 15 had painful neuropathy induced by spinal nerve ligation (SNL). All SNL animals demonstrated hyperalgesic responses, whereas no animals in the Control group did. The frequency of hyperalgesia responses from noxious punctate mechanical stimulation in SNL rats (32 ± 3%) was greater than in control rats (0 ± 0%; *P* < 0.001). All SNL animals used in this study had greater than 20% hyperalgesia responses (at least 6 responses to a total of 30 pin stimulations from 3 sensory test sessions). Within a single testing session, we see no pattern of accumulating sensitivity or accommodation to the stimuli. The anatomical accuracy of the SNL surgery was confirmed at the time of tissue harvest in all SNL animals.

### Both DRG neurons and satellite cells exhibit σ1R immunofluorescence

**Figure 1:**
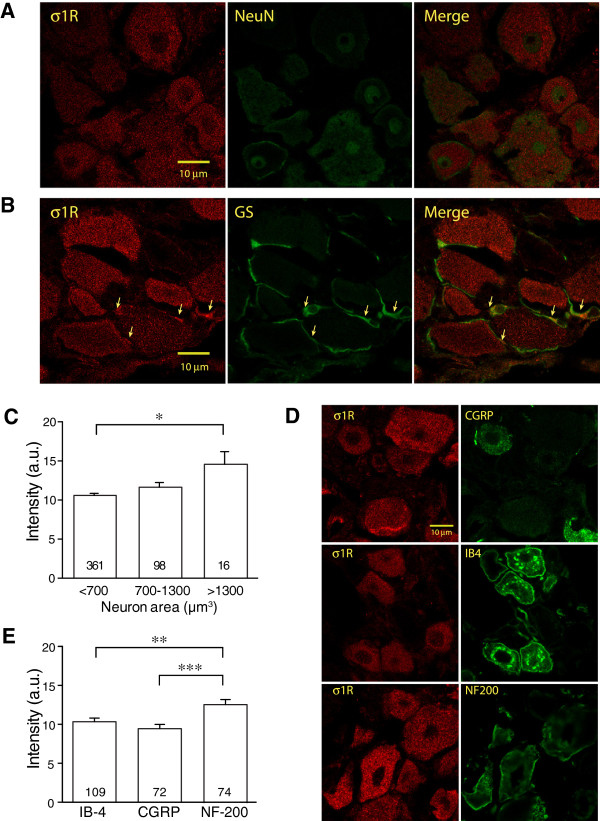
**σ1 Receptor (σ1R) in various Control dorsal root ganglion (DRG) cell populations. (A)** Staining for σ1R was uniformly distributed within cytoplasmic areas of cells identified as neuronal profiles through co-staining with NeuN. No specific σ1R staining was found in neuronal nuclei. **(B)** σ1R was also found in cellular components identified as satellite glial cells by their expression of glutamine synthase (GS). Identically placed arrows in the left and middle panels indicate cellular areas clearly identifiable as satellite glial cell cytoplasm that expresses σ1R. **(C)** Classification of control DRG neurons by size (<700, 700-1300 and >1300 μm^3^) showed greater σ1R intensity (arbitrary units, recorded using standardized image acquisition) in large neurons compared to others. **(D)** σ1Rs expression is compared in neuronal subtypes identified by their expression of calcitonin-gene related peptide (CGRP), isolectin B-4 (IB4), and neurofilament-200 (NF-200). **(E)** Higher fluorescent intensity for σ1Rs was found in the subpopulation of control neurons expressing NF-200. Mean ± SEM; number in bars represents the sample size; *p < 0.05, **p < 0.01, ***p < 0.001.

We first used immunohistochemical (IHC) methods to identify if σ1R protein is present in the DRG. Fluorescent images of σ1R staining in DRG sections revealed the presence of σ1R in neuronal somata in a punctate distribution in the cytoplasm (Figure 1A,B), consistent with its location in the ER [1,43]. Double staining for σ1R combined with NeuN, a neuron-specific marker, revealed expression of σ1R in all neurons (Figure 1A). Using glutamine synthetase [44,45] as a marker for astrocyte-like SGCs [46], sections also demonstrated expression of σ1R in SGCs (Figure 1B). This finding is consistent with prior reports of the presence of σ1R in glial cells such as astrocytes and Schwann cells [3,47]. When classified into different neuronal subpopulations by size, large neurons comparatively higher levels of σ1R expression (Figure 1C). This suggests that there may be neuron subtype-specific expression of σ1R. Accordingly, we compared σ1R expression in neuronal groups characterized by expression of CGRP, a marker of small unmyelinated peptidergic primary sensory neurons, by binding of IB4, a marker of small unmyelinated non-peptidergic sensory neurons, and by expression of NF200, a marker of large, myelinated neurons [48,49]. This analysis confirmed comparatively higher σ1R expression in the NF200 group (Figure 1D, E), consistent with their larger size.

### Effect of injury on σ1R Expression in DRG

**Figure 2:**
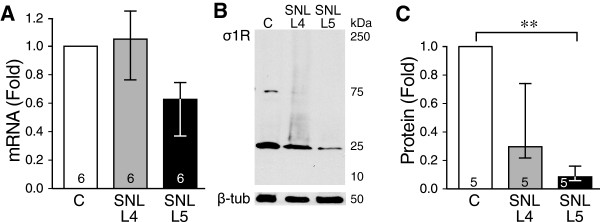
**Effect of injury on σ1 Receptor (σ1R) expression in dorsal root ganglia (DRG). (A)** Transcript measured by quantitative real time PCR showed no significant differences in σ1R level after spinal nerve ligation (SNL) in both the axotomized 5th lumbar (L5) dorsal root ganglion and the adjacent 4th lumbar (L4) ganglion, compared to control (C) ganglia. **(B)** Protein measurement by Western blot showed a dominant band at the expected molecular weight that was decreased in both L4 and L5 ganglia after SNL compared to control DRGs. β-Tubulin 1 (β-Tub) was used as loading control. **(C)** Summary data of Western blot showed a significant decrease of σ1R protein expression in axotomized L5 DRG. Both graphs show Median and interquartile range for fold difference from normalized controls; numbers in bars represents the sample size; **p < 0.01.

Transcripts for σ1R have previously been found in neuronal tissues [50,51], but their presence has not been examined in peripheral sensory neurons. We identified σ1R at the transcript level in DRG lysates (Figure 2A), confirming the IHC findings of its expression there. As an initial examination of the effect of injury on sensory neuron σ1R expression, we also examined lysates from animals that developed painful neuropathy following SNL. Examination of transcript level (Figure 2A) did not show any significant changes. However, Western immunoblotting of lysates using a commercially available antibody (Invitogen) demonstrated significantly down-regulated σ1R protein expression in SNL L5 DRGs compared to Control, while neighboring SNL L4 DRGs showed an intermediate level of σ1R protein expression (Figure 2B,C). These Western findings were duplicated using another σ1R antibody (gift from Drs. T. P. Su and T. Hayashi at NIDA; data not shown).

### Effect of injury on σ1R immunofluorescence in subpopulations of DRG neurons

**Figure 3:**
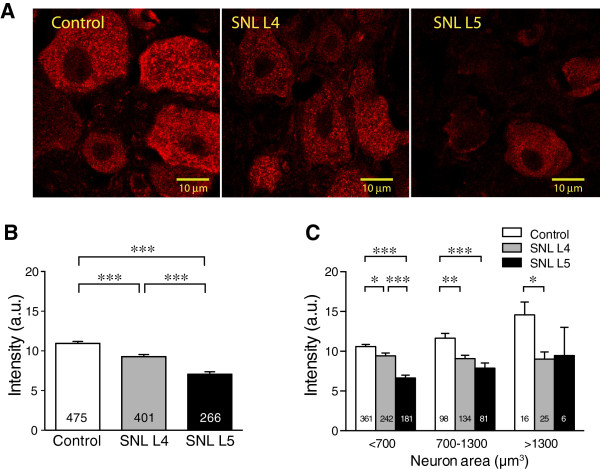
**Effect of injury on σ1 receptor (σ1R) expression in sensory neurons. (A)** Decreases of σ1 receptor (σ1R) immunofluorescence intensity were found in both spinal nerve ligation (SNL) 4th lumbar (L4) and 5th lumbar (L5) neurons compared with that in Control animals. **(B)** Summary data showed decrease of intensity of staining in SNL L4 and L5 neurons. Number in bars represents the n for neurons. **(C)** Classified by neuronal size (<700, 700-1300 and >1300 μm^3^), SNL L4 DRG neurons showed lower σ1R intensity (arbitrary units, recorded using standardized image acquisition) than Control in all three size groups, and SNL L5 neurons in the small and medium size groups showed lower σ1R intensity than Controls. The data for Control groups are the same as shown in Figure 1C. Mean ± SEM; *p < 0.05, **p < 0.01, ***p < 0.001.

To further evaluate the effect of injury on σ1R expression, we quantified IHC images from DRG sections following SNL (Figure 3A). Immunofluorescent intensity of σ1R was attenuated in both axotomized SNL L5 DRGs and, to a lesser extent, in the neighboring SNL L4 DRG (Figure 3B). The finding is consistent with our Western blotting result (Figure 2), in which σ1R protein expression decreased more in SNL L5 than in SNL L4 DRGs. This pattern was found in small and medium size groups (Figure 3C), although large neurons failed to show an effect on the SNL L5 group, which were few in number.

**Figure 4:**
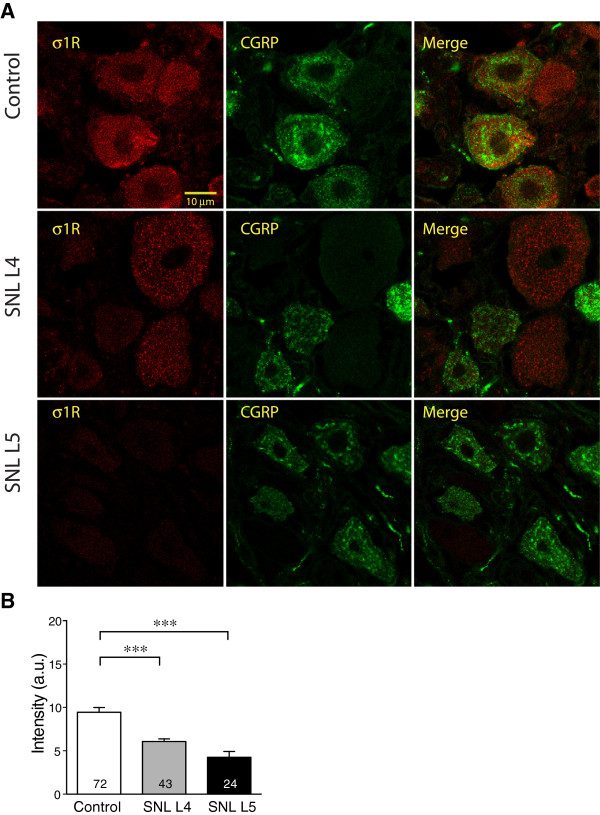
**Effect of injury on σ1 receptor (σ1R) expression in sensory neurons identified by calcitonin-gene related peptide (CGRP) staining. (A)** Staining of σ1R was distributed within cytoplasmic areas of all CGRP co-expressing neurons. σ1R immunofluorescence intensity was decreased in both spinal nerve ligation (SNL) 4th lumbar (L4) and 5th lumbar (L5) neurons compared with Control. **(B)** Summary data showed decrease of intensity of σ1R staining in CGRP-positive SNL L4 and L5 neurons. Mean ± SEM; number in bars represents the sample size; ***p < 0.001.

**Figure 5:**
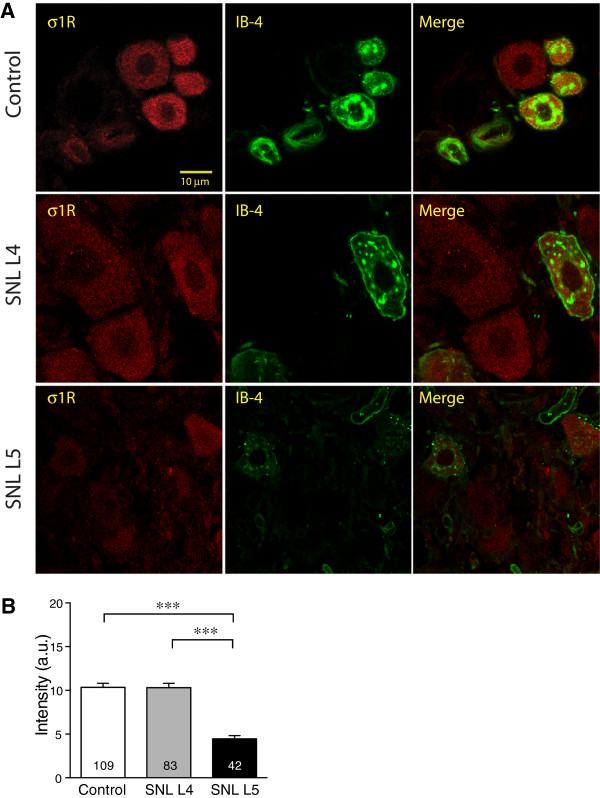
**Effect of injury on σ1 receptor (σ1R) expression in sensory neurons identified by IB4 staining. (A)** Staining of σ1R was distributed within cytoplasmic areas of all IB4 co-expressing neurons. σ1R immunofluorescence intensity was decreased in spinal nerve ligation (SNL) 5th lumbar (L5) neurons compared with Control. **(B)** Summary data showed decrease of intensity of σ1R staining in IB4-positive SNL L5 neurons. Mean ± SEM; number in bars represents the sample size; ***p < 0.001.

**Figure 6:**
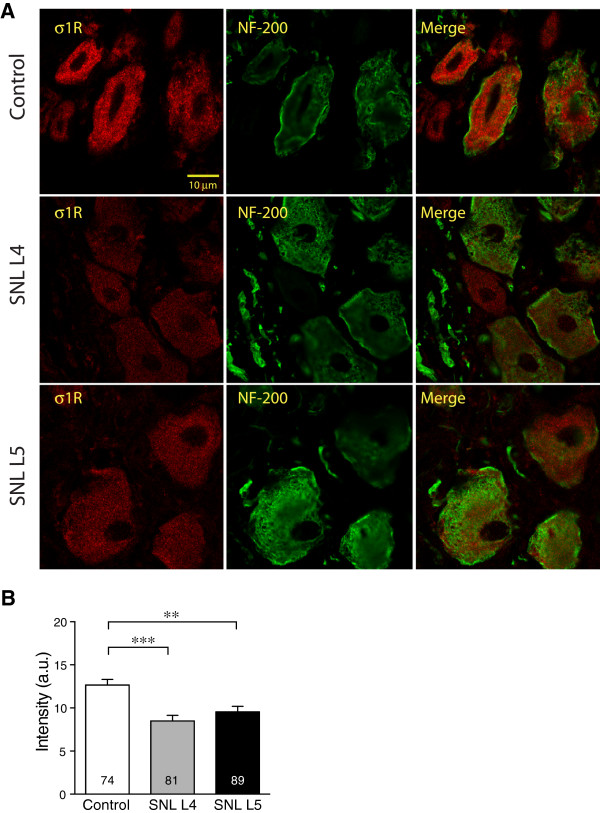
**Effect of injury on σ1 receptor (σ1R) expression in sensory neurons identified by neurofilament-200 (NF-200) staining. (A)** Staining of σ1R was distributed within cytoplasmic areas of all NF-200 co-expressing neurons. σ1R immunofluorescence intensity was decreased in both spinal nerve ligation (SNL) 4th lumbar (L4) and 5th lumbar (L5) neurons compared with Control. **(B)** Summary data showed decrease of intensity of σ1R staining in IB4-positive SNL L4 and L5 neurons. Mean ± SEM; number in bars represents the sample size; **p < 0.01, ***p < 0.001.

For the neuronal subpopulation labeled with CGRP (Figure 4A), we found that σ1R expression decreased not only in SNL L5 but also in neighboring uninjured L4 DRG neurons, compared to Control (Figure 4B). IB4 showed plasma membrane and perinuclear staining (Figure 5A), consistent with a previous description [52]. Double staining for σ1R with IB4 revealed that expression of σ1R decreased in SNL L5 DRGs but not in SNL L4 neurons (Figure 5B). Double staining for σ1R with NF200 (Figure 6A) showed decreased σ1R expression in both L4 and L5 DRGs after SNL, compared to Control (Figure 6B).

### Effect of injury on σ1R immunofluorescence in DRG satellite glial cells

**Figure 7:**
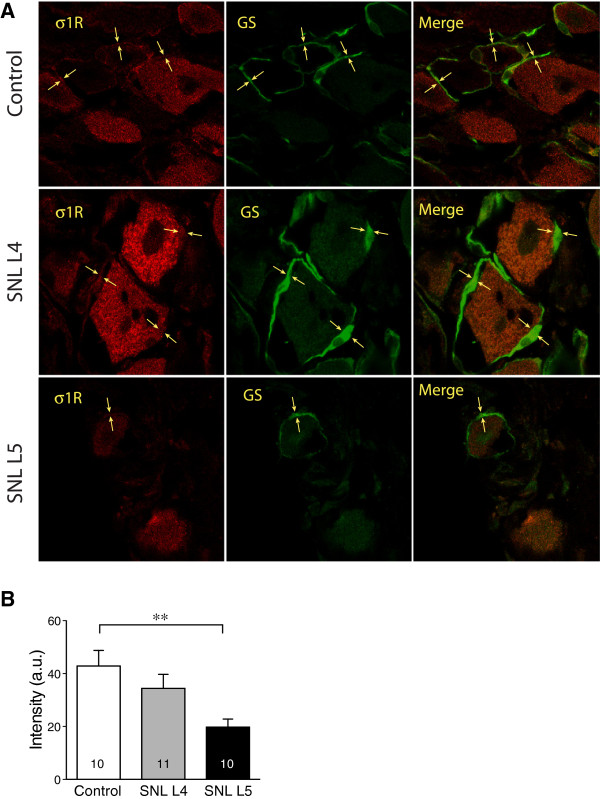
**Effect of injury on σ1 receptor (σ1R) expression in satellite glial cells identified by glutamine synthase (GS) staining. (A)** Intensity of σ1R was decreased in cytoplasmic areas of satellite glial cells in 5th lumbar (L5) DRGs after spinal nerve ligation. **(B)** Summary data for average intensity in satellite glial areas showed decrease of intensity of σ1R staining in GS-positive glial cells in SNL L5 dorsal root ganglia. Mean ± SEM; number in bars represents the section sample size; **p < 0.01.

Using immunostaining of glutamine synthetase to detect satellite glial cells, we observed diminished σ1R immunofluorescent intensity within the satellite glial cells of the L5 DRG following SNL (Figure 7A,B).

## Discussion

Several key findings emerge from our examination of σ1R expression in control and injured sensory neurons. We demonstrated that σ1Rs were present in both sensory neurons and SGCs. In line with previous reports [5,43,53,54], we found that σ1Rs are dispersed throughout the cytoplasm of DRG neurons in all size and histochemical subgroups. Expression of the σ1R protein decreases in both axotomized L5 neurons as well as neighboring L4 neurons, and this decrease occurred in all neuronal subpopulations. These observations confirm the presence of σ1R in the DRG, which was previously suspected on the basis of ligand binding studies [53,55]. Furthermore, our findings also support a role for primary sensory neuron σ1Rs in pain generation after injury.

In the present study, we determined the average σ1R expression in relevant DRG neuronal subgroups and the effect of SNL. In prior IHC analysis by Hammond et al. [52], it was shown that SNL also affects the number of neurons in these subgroup populations. Specifically, axotomy decreases the number of neurons in both the CGRP and IB4 subgroups in L5 DRGs, whereas SNL L4 neurons are unaffected [52]. These populations recover over the ensuing 20 weeks, and partial recovery occurs by the 3-week time point that we used in the present study. This downward shift in neuron population size, when combined with our observation of decreased σ1R expression per neuron, could amplify the functional effect of depression of σ1R in neurons of the CGRP and IB4 subgroups (typically small nociceptors). Our data do not provide any insight into the cause of depressed σ1R levels after nerve injury. However, it is possible that accelerated axonal transport of σ1R from the sensory neuron soma may account for receptor depletion there. Specifically, σ1R bound to the ER membrane is activated by ER stress, such as occurs with ER Ca^2+^ depletion that follows nerve injury [35]. Thereupon, σ1R is relocated to targeted cell compartments [38,56,57], which may include non-somatic regions.

We found that σ1R expression was depressed in SNL L4 DRGs, although not to the same extent as in axotomized L5 neurons and not in the IB4 population. It has been shown that L5 axotomy results in changes of a variety of signaling peptides and cellular function in the uninjured L4 DRG neurons [58,59]. In common with these phenotypic shifts, decreased σ1R expression in SNL L4 neurons may result from inflammatory processes triggered by the degeneration of axotomized distal segments of L5 neurons within the distal sciatic nerve [60-62].

We must consider what our present findings may indicate for the role of σ1R in pain processing. It is clear that σ1R activation potentiates nociceptive responses under both baseline conditions and in the setting of facilitated pain models [20,22,24,63,64]. In the specific setting of nerve injury, responses to mechanical and thermal stimuli are potentiated by σ1R agonists and attenuated by σ1R antagonists or genetic elimination of σ1R [65]. Implicated signaling pathways include nitric oxide, PKC, PKA, and NMDA receptor in the spinal cord [24-29]. One possible explanation of these events would be injury-induced overexpression of σ1R. Our results, however, clearly indicate that σ1R expression is down-regulated in primary sensory neurons in the chronic phase after axotomy, although we did not examine earlier time points. In contrast to our DRG findings, spinal cord dorsal horn homogenates show elevated σ1R protein expression early (3 days) after sciatic nerve injury [25], but since expression returns to control level by day 7, a shift in dorsal horn σ1R levels is also unlikely to contribute chronic neuropathic pain. It therefore appears that the σ1R activation and blockade influence pain signaling either through the relatively unaffected L4 neurons, or that the σ1R remaining in the axotomized population is sufficient for these modulators to alter pain generation. Likewise, there may be a pathogenic role for σ1R in neuropathic pain if levels of endogenous neurosteroid agonists such as dehydroepiandrosterone [66] and pregnenolone increase sufficiently to produce elevated σ1R effects despite lowered σ1R expression.

Recent studies have shown that σ1Rs are present in oligodendroglia and astrocytes in the CNS [4,47,67,68] and in Schwann cells of the sciatic nerve [3]. Since SGCs in the DRG play a similar role as astrocytes in CNS [46] and come from the same embryological origin as Schwann cells [69], our finding of σ1R in SGCs is consistent with these previous reports. After ischemic brain injury, σ1Rs are upregulated in reactive astrocytes as well as neurons [67], which reduces glutamate accumulation and inhibits interleukin-1β expression and microglial migration to abate ischemia-induced inflammatory reaction [70,71] (reviewed in [7]). Our data show that peripheral nerve injury differs from ischemic brain injury since σ1R decreases in the ganglia in which axotomized neurons reside. This may deprive such neurons of the evident protective effects of σ1R modulation of inflammatory processes and indirectly contribute to the generation of chronic pain after nerve injury.

## Conclusions

Our findings indicate that σ1Rs are present in sensory neurons, thus supporting these neurons as a possible site of action in enhancing pain after intrathecal σ1R activation and reducing pain with σ1R blockade [25,27,72]. After peripheral nerve injury, σ1R expression is decreased in axotomized L5 and to a lesser extent in neighboring L4 DRG neurons in all tested neuronal subpopulations, which eliminates injury-induced change of neuronal receptor expression as a pathogenic mechanism. Overexpression of endogenous σ1R agonists may account for sensitivity of neuropathic pain to σ1R blockade, and loss of glial σ1R may be a contributing factor.

## Methods

### Animals

All methods and use of animals were approved by the Medical College of Wisconsin Institutional Animal Care and Use Committee. Male Sprague-Dawley rats (Taconic Farms Inc., Hudson, NY) were housed individually in a room maintained at 22 ± 0.5°C and constant humidity (60 ± 15%) with an alternating 12 hr light-dark cycle. Food and water were available *ad libitum* throughout the experiments.

### Injury model

Rats weighing 150 to 180 g were subjected to SNL modified from the original technique [73]. Specifically, rats were anesthetized with 2% isoflurane in oxygen and the right paravertebral region was exposed. The L6 transverse process was removed, after which the L5 and L6 spinal nerves were ligated with 6-0 silk suture and transected distal to the ligature. To minimize non-neural injury, no muscle was removed, muscles and intertransverse fascia were incised only at the site of the two ligations, and articular processes were not removed. The muscular fascia was closed with 4-0 resorbable polyglactin sutures and the skin closed with staples. Control animals received skin incision and closure only. After surgery, rats were returned to their cages and kept under normal housing conditions with access to pellet food and water *ad lib*.

### Sensory testing

We measured the incidence of a pattern of hyperalgesic behavior that we have previously documented to be associated with conditioned place avoidance [74,75]. Briefly, on day 10, 12, and 17 after surgery, right plantar skin was touched (10 stimuli/session) with a 22G spinal needle with adequate pressure to indent but not penetrate the skin. Control animals respond with only a brief reflexive withdrawal, whereas rats subjected to SNL may display a complex hyperalgesia response that includes licking, chewing, grooming and sustained elevation of the paw, which peaks in frequency by day 17. Because of day-to-day variability in individual rat responses, the average frequency of hyperalgesia responses over the 3 testing days was tabulated for each rat. After SNL, only rats that displayed a hyperalgesia-type response after at least 20% of stimuli were used further in this study.

### Quantitative real-time PCR analysis

Total RNA was isolated from the homogenized L4 and L5 DRG of control animals (n = 6), and separately from the L4 and L5 DRGs of SNL rats (n = 6) harvested 21 days after surgery, following the manufacturer’s (Invitrogen, Carlsbad, CA) instructions using Trizol reagent (from aqueous phase). After DNase treatment, cDNA was synthesized from amounts of RNA that were standardized for each experiment ranging from 152 to 232 ng for different experiments, using random hexamer primers (Superscript III first strand synthesis kit; Invitrogen, Grand Islands, NY). Quantitative real time PCR (qPCR) analysis was carried out using IQ Syber Green supermix (Biorad Laboratories, Hercules, CA) and specific primers to quantify the cDNA levels of the σ1R genes (forward primer (FP), 5’-CCTGCTGGCATTCGGGGCTC-3’; reverse primer (RP), 5’-TCACAGCTGCAGGCGAACGG-3’; NCBI reference sequence, NM_030996.1). A preliminary gel showed that PCR products had the expected molecular weight and a melting curve was run in each sample to confirm single product in every run (data not shown). Normalization was carried out using the geometric mean of two reference genes, glyceraldehyde 3-phohphate dehydrogenase (GAPDH; FP, 5′-AGACAGCCGCATCTTCTTGT-3′; RP, 5′-TGATGGCAACAATGTCCACT-3′) and mitogen-activated protein kinase 6 (MAPK6; FP, 5′-TAAAGCCATTGACATGTGGG-3′; RP, 5′-TCGTGCACAACAGGGATAGA-3′), which were chosen for their stability in the context of SNL injury [76]. For each sample, two inter-run determinations were averaged and the fold differences in expression (SNL L4, and SNL L5 DRG) were compared to that of the Control samples using the comparative C_T_ method. Statistical analysis of ∆∆C_T_ values used for σ1R, normalized with MAPK6/GAPDH, was performed using Kruskal-Wallis test (*p* = 0.073) with *Post hoc* by Dunn’s test. Graphs were plotted using 2^-ΔΔCT^ values for representation of σ1R gene expressions in different groups.

### Western blot analysis

Total protein was isolated from individual L4 and L5 DRGs of Control animals, and separately from L4 and L5 DRGs of SNL animals. Harvested ganglia were homogenized in 150 μl of RIPA lysis buffer containing protease inhibitors (Roche Diagnostics, Indianapolis, IN) and phosphatase inhibitors (Thermo Scientific, Rockford, IL), and incubated on ice for 30 minutes. Lysates were spun down at 14000 rpm for 10 min at 4°C. The supernatant was used for protein estimation using Pierce bicinchoninic acid protein assay kit (Thermo Scientific, Rockford, IL). Equal amounts of protein (30 μg) were separated on 4-15% sodium dodecyl sulphate polyacrylamide gel electrophoresis gel (SDS-PAGE; Bio-Rad, Hercules, CA) and transferred onto a polyvinylidene fluoride membrane. After blocking with 5% milk in TBS-T (Tris-buffered saline plus 0.1% Tween 20), blots were sequentially probed overnight at 4°C with one of two anti-σ1R rabbit polyclonal antibodies, either a commercial product (1:500, Invitrogen, Camarillo, CA, catalog number 42-3300; Lot No. 797152A, polyclonal affinity-purified antibody raised against a synthetic peptide derived from the C-terminus region (amino acid 139-155) of the rat σ1R), or another that was a kind gift from Drs. Su and Hayashi of NIDA (1:1000, Lot No. 6405, polyclonal affinity-purified antibody raised against amino acid 52-69 from the C-terminus of rat σ1R) [6,77]. Loading control was used anti-β-tubulin I mouse monoclonal antibody (1:5000, Sigma-Aldrich, St. Louis, MO, catalog number T7816, Lot No. 088 K4795, monoclonal antibody raised against a synthetic peptide corresponding to the C-terminus sequence of β-tubulin isotype I, conjugated to BSA) [45]. Three 15 min washes in TBS-T followed before probing with secondary antibody of horseradish peroxidase-conjugated goat anti-rabbit antibody (1:2000, Pierce, Rockford, IL). Enhanced chemiluminescence (ECL plus; Amersham, Piscataway, NJ) was used to detect protein bands. Western blot restore stripping buffer (Thermo Scientific, Rockford, IL) was used to strip antibodies from the membrane. The bands obtained were quantified using ImageJ (U.S. National Institutes of Health, Bethesda, MD) program, and β-tubulin I was used to normalize the protein loading. The normalized values (to β-tubulin I) of Control, SNL L4 and SNL L5 isoform were used for the statistical analysis. Although the antibody from NIDA showed a strong expression band at the expected molecular weight (25 kDa), the blot also showed non-specific bands as well (data not shown). The more selective Invitrogen antibody was used for immunohistochemistry.

### Immunohistochemistry

Twenty-one days post-surgery, the Control, SNL L4 and SNL L5 DRGs were harvested and cryoprotected in 4% paraformaldehyde with 15% sucrose in 0.1 M PBS for 1 h, followed by incubation in 30% sucrose 0.1 M PBS overnight [49]. Tissues embedded in Tissue-Tek optimal cutting temperature compound (Ted Pella, Inc., Redding, CA) were sectioned (10 μm) with a Leica cryostat (Jung CM 1800; Vienna, Austria), plated onto subbed slides (Superfrost Plus Gold; Fisher Scientific, Pittsburgh, PA), and post-fixed in 4% paraformaldehyde with 4% sucrose for 10 min. After blocking with 10% normal goat serum for 1 h at room temperature, the tissue was incubated overnight with anti-σ1R rabbit polyclonal antibody (1:100, Invitrogen, same antibody used Western) [77]. After four washes with PBST, sections were incubated with Alexa Fluor 568 Goat anti-rabbit antibody (1:500; Invitrogen, Camarillo, CA) for 2 h. To determine co-localization of σ1R with the neuron-specific nuclear protein (NeuN), neurofilament 200 (NF-200), CGRP, and glutamine synthetase, the sections were washed four times with PBST followed by incubated with anti-NeuN mouse monoclonal antibody (1:100, Millipore, Billerica, MA, catalog number MAB377, Lot No. LV1616015, monoclonal antibody raised against purified cell nuclei from mouse brain) [45], anti-NF-200 mouse monoclonal antibody (1:1000, Abcam, Cambridge, UK, catalog number ab28029, monoclonal antibody raised against a non phosphorylated epitope from 200kD Neurofilament Heavy of most mammalian species) [49], anti-CGRP mouse polyclonal antibody (1:50, Santa Cruz Biotechnology, Santa Cruz, CA, catalog number SC-28920, Lot No. H1407, epitope corresponding to amino acid 81-128 mapping at the C-terminus of CGRP of human origin) [49], or anti-glutamine synthetase rabbit polyclonal antibody (1:500, Santa Cruz Biotechnology, Santa Cruz, CA, catalog number SC-6640, Lot No. H1407, polyclonal affinity purified antibody raised against a peptide mapping at the C-terminus of glutamine synthetase of human origin) [45] for 2 h. Following four washes with PBST, sections were incubated with Alexa Fluor 488 goat anti-mouse antibody or Alexa Fluor 488 goat anti-rabbit antibody (1:1000; Molecular Probes, Camarillo, CA) for 1 h. To determine co-localization of σ1R with IB4, sections were incubated with Alexa Fluor 488 conjugate IB_4_ (1:50, Invitrogen, Camarillo, CA, catalog number I21411, Lot No. 743633,) [78] after four washes with PBST. Sections were washed four times with PBST and covered with Prolong Gold Antifade mounting medium (Invitrogen, Camarillo, CA). Sections were examined by confocal microscopy. The expression level of σ1R protein was represented by the image intensity that was captured using standardized camera parameters (Metamorph, Downingtown, PA), and cell area was determined by outlining the neuronal profile by excluding its nucleus. A neuron was considered positive when intensity in cells of interest was two-fold greater than in background in sections incubated without targeting primary antibody. At least 3 sections from each DRG were randomly chosen for fluorescence intensity measurement, except in a case of SNL L5 DRG with σ1R/CGRP double staining, for which 2 sections were evaluated. The fluorescence intensity of all cells in the section was quantified. The individual who measured the fluorescence intensity was not completely blinded to the treatment due to obvious markers of axotomy such as eccentric location of the nucleus. Average fluorescence intensity of neurons was measured in traced cytoplasmic areas of interest after subtracting background fluorescence [49,79]. Satellite glial cell σ1R average intensity was derived by creating a mask from a thresholded image of glutamine synthetase immunofluorescence to isolate areas of interest in the image of σ1R immunofluorescence using Photoshop (Adobe Systems Inc., New York City, NY).

### Statistical analysis

Prism (version 6.1, GraphPad Software, Inc., San Diego, CA) was used to perform paired or unpaired Student’s t-test or one-way ANOVA. Data were derived from at least 3 DRGs for every group. Non-parametric Kruskal-Wallis test with *Post hoc* by Dunn’s test was used for analyzing the influence of injury on σ1R in qPCR and Western immunoblot experiments. In IHC experiments, main effects identified by ANOVA were further analyzed by Tukey’s test (for comparisons between all groups) to compare relevant means. Data are reported as median with interquartile range for qPCR and Western immunoblot experiments, and as mean ± SEM for IHC experiments. A *P* value less than 0.05 was considered significant.

## Abbreviations

SNL: Spinal nerve ligation; CNS: Central nervous system; DRG: Dorsal root ganglion; SGC: Satellite glial cells; σ1R: Sigma-1 receptor; IB4: Isolectin-4; CGRP: Calcitonin gene-related protein; NeuN: Neuron-specific nuclear protein; NF-200: Neurofilament 200; IHC: Immunohistochemical.

## Competing interests

The authors declare that they have on competing interests.

## Authors’ contributions

BML designed quantitative real time PCR experiment, acquired, analyzed and interpreted data and also contributed to western blotting and IHC studies; DM contributed to confocal microscope study in analyzing and interpreted IHC data; VZ contributed to IHC studies; QBT contributed to western blotting and IHC study designs; QH participated in interpretation of the data, and revising the manuscript; HEW contributed to the conception and design of the studies, interpretation of the data, writing and revising the manuscript. All authors read and approved the final manuscript.
